# Dietary Adherence of Saudi Males to the Saudi Dietary Guidelines and Its Relation to Cardiovascular Diseases: A Preliminary Cross-Sectional Study

**DOI:** 10.3390/jcdd6020017

**Published:** 2019-04-04

**Authors:** Areej Ali Alkhaldy, Reem Saleh Alamri, Rozana Khalid Magadmi, Nrvana Yasser Elshini, Rania Abd El Hamid Hussein, Kamal Waheeb Alghalayini

**Affiliations:** 1 Clinical Nutrition Department, Faculty of Applied Medical Sciences, King Abdulaziz University, P.O. Box 80215, Jeddah 21589, Saudi Arabia; reemsalamri@gmail.com (R.S.A.); rozana-magadmi@hotmail.com (R.K.M.); nrvanaalshini@gmail.com (N.Y.E.); rahussein2002@yahoo.com (R.A.E.H.H.); 2 Internal Medicine Department, Gamal Abd El Nasser Hospital, Health Insurance Authority, Alexandria 21516, Egypt; 3 Department of Medicine, Faculty of Medicine, King Abdulaziz University, P.O. Box 80215, Jeddah 21589, Saudi Arabia; kalghalayini@kau.edu.sa

**Keywords:** dietary intake, nutrition, cardiovascular disease

## Abstract

Cardiovascular disease (CVD) is a major public health problem in Saudi Arabia. Dietary intake plays a major role in CVD incidence; however, the dietary intake status in Saudi nationals with CVD is unknown. We aimed to investigate whether the dietary patterns of Saudi males, using the Saudi dietary guidelines adherence score, in parallel with the measurement of a selective number of cardiovascular disease-related biomarkers, are contributing factors to CVD risk. Demographics, dietary adherence score, and blood biomarker levels were collected for 40 CVD patients and forty non-CVD patients. Fasting blood glucose (*p* = 0.006) and high-density lipoprotein levels (*p* = 0.03) were significantly higher in CVD patients. The adherence score to the Saudi dietary guidelines was not significantly different between the CVD and non-CVD patients; however, the specific adherence scores of fruit (*p* = 0.02), olive oil (*p* = 0.01), and non-alcoholic beer (*p* = 0.02) were significantly higher in the non-CVD patients. The differences in CVD family history (*p* = 0.02) and adherence scores to specific groups/foods between the CVD and non-CVD patients may contribute to CVD risk in Saudi males. However, as the sample size of this study was small, further research is required to validate these findings.

## 1. Introduction

Cardiovascular disease (CVD) is considered a major public health problem in Saudi Arabia [1,2]. with an estimated 46% of all deaths attributed to CVD, and a 36% higher death rate in men compared to women [1]. The risk factors of CVD are characterized as modifiable factors such as diet, physical activity, obesity, and smoking, or non-modifiable factors such as aging, family history, and ethnicity [3,4,5]. The most important modifiable risk factor for CVD is diet [6,7,8,9]. Studies showed that a diet high in fruits, vegetables, whole grains as the major source of carbohydrates, and non-hydrogenated unsaturated fats as the main form of dietary fat, with adequate omega-3 fatty acids (monounsaturated fatty acid and polyunsaturated fatty acid), may reduce the risk of CVD [9,10,11,12,13]. Vitamins, minerals, fiber, and phenolic compounds are the main protective components found in fruit, vegetables, and whole grains with a functional role in reducing oxidative stress, inflammation, blood pressure, and improving insulin sensitivity and the lipoprotein profile [14,15,16]. In contrast, a diet containing a high intake of saturated fats, and refined and processed carbohydrates is linked with an increased CVD risk as a result of raised levels of blood glucose, total cholesterol, and low-density lipoprotein (LDL) cholesterol [17,18,19,20,21,22].

However, it is important to note that most of these nutritional studies evaluated the intake of a single nutrient, or small number of nutrients or food items in relation to risk of CVD. As individuals do not eat a single food or isolated nutrient, many researchers argued the importance of considering a holistic approach, investigating dietary patterns (combination of nutrients) rather than nutrient-based studies, when assessing health consequences [23,24,25]. Moreover, diet has a synergistic effect as it is a complex mixture of nutrients (within-food or across-food combinations), which could induce antagonistic effects on optimal health [23,24,25].

Dietary guidelines are a useful tool in public health policy, which can help reduce risk and prevent non-communicable diseases. Studies on dietary guideline adherence demonstrated their effectiveness in reducing the risk of disease, including CVD in multiple countries [24,26,27].

Current CVD research is dominated by studies that were conducted on Western populations with a paucity of research investigating the link between CVD and diet in Saudi Arabia [28,29]. Furthermore, to our knowledge no study used a holistic approach to consider the association of adherence to Saudi dietary guidelines with risk of CVD. We hypothesized that the non-adherence to the Saudi dietary guidelines could increase the risk of CVD in Saudi males. 

## 2. Material and Methods

### 2.1. Study Design and Participants

A cross-sectional design study was performed at King Abdulaziz University Hospital (KAUH) in Jeddah, Saudi Arabia. Patients were recruited from the medical ward, surgical ward, and coronary care unit at KAUH. The inclusion criteria for CVD patients consisted of male cardiac patients, aged between 30–80 years old, as the prevalence of CVD among Saudi population increases after 30 years old [30]. The inclusion criteria for the non-CVD patients included male patients, in the same age range, who were free of CVD and had been admitted to the hospital for minor clinical conditions, including abdominal pain, eye surgery, and fever. Patients with liver, kidney, or respiratory disease, or any type of cancer diagnosis were excluded from recruitment to either group. Ethical approvals were obtained from the Faculty of Medicine Research Committee at King Abdulaziz University (Reference no. 307-14). All patients gave informed written consent.

### 2.2. The Saudi Dietary Guidelines

The message of the Saudi dietary guidelines is to follow a healthy diet including variety, balance, and moderation [31]. The main goals of these guidelines are (1) to improve health by promoting healthy eating options and encouraging physical activities; (2) to promote valuable food that high in nutrients such as foods rich in protein, fiber, vitamins, and minerals, and reduce foods of poor nutritional value such as foods high in salt, sugars, saturated fats, and hydrogenated fat; (3) to support the normal growth and development of infants, children, and adolescents; (4) to decrease the diseases related to diet in the Saudi community; and (5) to support physical activity.

To communicate the recommended food groups and serving sizes, the Saudi dietary guidelines are graphically represented in the form of a palm tree with the food groups distributed in the trunk and leaves of the palm in proportion to their recommended level of intake. The largest food group of bread and cereals was placed in the bottom big leaf of the palm and represents the most important source of carbohydrates (6–11 servings/day). Vegetables (3–5 servings/day) and fruits (2–4 servings/day) come next as they are high in vitamins and minerals. Milk and its products (2–4 servings/day) are the third largest group, which are essential sources of protein and calcium. The smallest group constitutes meat and beans (2–4 servings/day), and they are considered as sources of protein. Fat and sugar were in the smallest upper leaves of the palm (representing lower quantities); this shows the need to minimize their intake. Water was also added to the healthy food palm, due to the hot weather of Saudi Arabia. As regular physical activity is essential, together with a balanced diet, the healthy food palm also recommends individuals to exercise for 30–60 min daily according to the individual’s health status [31].

### 2.3. The Saudi Dietary Guideline Score

The score of adherence was given according to the Saudi dietary guidelines [31]. The ratings of the consumption of each food group (from 0 to 5 or the reverse) was adapted from Panagiotakos et al. (2006) [24]. The dietary adherence score included non-refined cereals and bread (whole bread, rice, pasta, and other grains), fruit, vegetables, legumes, fish, olive oil, non-alcoholic beer, meat and meat products, poultry, full-fat dairy products, sweets, and oils. 

For the intake of food items assumed to be close to the Saudi dietary guidelines or higher (non-refined cereals, fruits, vegetables), we allocated a score of 0 when the individual stated no consumption, a score of 1 when they stated consumption of 1–4 servings/month, a score of 2 for 5–8 servings/month, a score of 3 for 9–12 servings/month, a score of 4 for 13–18 servings/month, and a score of 5 for more than 18 servings/month. Moreover, we included legumes, fish, and olive oil in this group after separating them from the meat and oil groups. Originally excluded from the Saudi guidelines, non-alcoholic beer was also added due to its health benefits for heart disease [32]. In contrast, for the intake of food items assumed to be limited in Saudi dietary guidelines (i.e., rare or monthly intake; meat and meat products, poultry, and full-fat dairy products), we allocated the scores on a reverse scale (i.e., 5, when individuals stated no intake, to 0, when they stated almost daily intake). Hence, the scores ranged from 0 to 60. Higher values of score show better adherence to the Saudi dietary guidelines (Table 1).

### 2.4. Procedure and Data Collection

An interview-administered survey consisting of four sections (demographics, anthropometrics, medical history, and a dietary assessment by food frequency questionnaire (FFQ)) was completed for each patient. 

### 2.5. Demographics and Medical History

Personal information, including date of birth, gender, marital status, any medical diagnoses, and family history of cardiovascular diseases (whether at least one first-degree relative had CVD) was collected from the hospital electronic system at KAUH. Data regarding the education level, employment status, and tobacco use was collected during the interview process.

### 2.6. Anthropometric Measurements

The anthropometric measurements, including height, weight, waist circumference (WC), and body mass index (BMI) were performed according to standard procedures and carried out in the patient’s ward. Patients were weighed in light clothing, without shoes, using a calibrated scale to the nearest 0.1 kg measured in kilograms. Height was measured to the nearest 0.1 cm. The BMI was computed as the fraction of weight to the squared height, with consideration for the cut-off for older adults (65 years old and older). The WC was taken at the level of the narrowest point between the lowest costal border and the iliac crest by the research team. 

### 2.7. Estimation of Habitual Dietary Intake

The dietary intake was assessed using a 60-item food frequency questionnaire (FFQ). For each food item, patients were asked how frequently it was consumed during the last year on a daily, weekly, or monthly basis, along with the portion sizes. Food intake was grouped into food groups (milk and dairy products, fruits, vegetables, meats, rice, breads, beverages, legumes, and sweets). The average servings of each item consumed per week were calculated using Microsoft Excel version 1808 (Redmond, WA, USA). 

### 2.8. Assessment of Blood Variables

Blood data were collected from the patients’ records for the lipid profile (high-density lipoprotein (HDL), low-density lipoprotein (HDL), total cholesterol (TC), and triglycerides (TG)); blood glucose (fasting blood glucose (FBG) and glycated hemoglobin (HbA1c)); hemoglobin (HGB) and hematocrit (HCT); cardiac enzymes (aspartate aminotransferase (AST), creatine kinase (CK), lactate dehydrogenase (LDH), and cardiac troponin I (CTN-I)); and electrolytes (sodium (Na), potassium (K), and chloride (Cl)), according to the hospital policy. 

### 2.9. Sample Size Calculation

This is a preliminary study to identify factors of interest and to develop a study protocol for a larger-scale study. No power calculation was conducted in this study due to the absence in Saudi literature of endpoints similar to the hypothesis proposed in this study. The data of this study serve as preliminary data to determine the needed sample size to achieve the aim of the study. We aimed to include 80 patients (40 with CVD and 40 without CVD disease).

### 2.10. Statistical Analysis

The statistical analysis was carried out using SPSS (version 23, SPSS, Inc, Chicago, IL, USA, 2015). A normality test was run on all data to determine if each dataset was well modeled by a normal distribution. Descriptive statistics are presented as medians and inter-quartile ranges (IQRs). 

Linear regression models were performed using the diet score as an independent variable, with age, smoking history, educational level, diabetic history, and family history of CVD as covariates. Systolic blood pressure, serum total cholesterol, LDL, HDL, triglycerides, waist circumference, and body mass index were utilized as outcome variables. The results were presented as *b*-coefficients and standard error of the coefficient. Bonferroni correction was used due to multiple comparisons. 

In addition, comparisons between the non-CVD patients and CVD patients were performed using a Mann–Whitney test for non-normally distributed data, and by paired *t*-test or two-sample *t*-test for normally distributed data. A chi-square test was used to compare the employment status and education level between the groups.

## 3. Results

### 3.1. Subject Characteristics

Eighty male patients were recruited for the study, aged between 30 and 80 years old. The demographic data for all individuals are presented in Table 2. There were no significant differences between non-CVD and CVD patients in terms of their age, education, or employment. However, there was a difference between non-CVD and CVD patients in terms of their family history, with significantly more CVD patients having at least one first-degree relative with CVD (*p* = 0.02; Table 3). The anthropometric characteristics, including blood pressure, weight, and BMI, were not significantly different between non-CVD and CVD patients (Table 4). The median BMI for both non-CVD and CVD patients was within the overweight cut-off range, with a value of 27.2 kg/m^2^ (IQR 23.4–31.1) for the non-CVD patients and 25.7 kg/m^2^ (IQR 23.4–27.7) for the CVD patients. The median waist circumference was within the normal range (< 102 cm) for both non-CVD (95.0 cm; IQR 85.8–100.0) and CVD patients (94.0 cm; IQR 90.0–98.3). 

### 3.2. Habitual Dietary Intake

The score of total adherence to the Saudi dietary guidelines was not significantly different between the non-CVD and CVD patients (Table 5); however, there were differences in adherence to dietary intake of some individual food groups. The specific adherence scores of fruit (*p* = 0.02), olive oil (*p* = 0.01), and non-alcoholic beer (*p* = 0.02) groups were significantly higher in the non-CVD patients (Table 5).

Moreover, we evaluated the effect of the Saudi diet on various health outcomes such as systolic blood pressure, serum cholesterol, low-density lipoprotein, high-density lipoprotein, triglycerides, waist circumference, and body mass index. Table 6 shows the results of multiple linear regression after adjusting for age, smoking history, educational level, diabetic history, and family history of CVD. There were only significant associations found between the total adherence to Saudi dietary guideline score and serum total cholesterol and LDL.

### 3.3. Blood Variables

There were no significant differences in the lipid profile measurements of total cholesterol, triglycerides, or LDL between the non-CVD and CVD patients (Table 7). However, the HDL was significantly higher in the CVD patients compared to the non-CVD patients (*p* = 0.03). The HDL was 1.1 mmol/L (IQR 1.0–1.5) in the CVD patients and 1.0 mmol/L (IQR 0.6–1.1) in the non-CVD patients. There was no difference in hematological profile, with the exception of the median fasting blood glucose level, which was raised in all patients (normal range <5.6 mmol/L), but significantly higher (*p* = 0.006) in the CVD patients (7.5 mmol/L; IQR 6.2–11.7) than the non-CVD patients (6.1 mmol/L; IQR 5.1–7.2). In terms of the electrolyte and cardiac enzyme levels (Table 7), all biomarkers were non-significant between the non-CVD and CVD patients, with the exception of the potassium level (*p* = 0.03), which was within normal range (3.6–5.2 mmol/L) for all patients, but significantly higher in the CVD patients 4.1 mmol/L (IQR 3.5–4.4) than the non-CVD patients 3.7 mmol/L (IQR 3.5–4.0). In contrast, the chloride level was also within the normal range for all patients (98–106 mmol/L) but significantly lower (*p* = 0.05) in the CVD patients (99.0 mmol/L; IQR 94.8–102.0) compared to the non-CVD patients (101.0 mmol/L; IQR 98.0–104.0).

## 4. Discussion

This study was performed to test the hypothesis that the dietary patterns of Saudis may play a role in increasing the risk of CVD. To our knowledge, this is the first study that assesses the dietary intake using the adherence score to the Saudi dietary guidelines and its relationship to CVD in Saudi males living in Jeddah. As such, the data obtained from this study may be considered an important preliminary step in gaining an increased understanding of variables in the Saudi population that may affect their risk of CVD.

This study found a number of significant differences between the non-CVD and CVD patients that align with previously published studies. Firstly, the present study found that a family history of CVD was significantly higher in the CVD patients. This indicates the strong effect of genetics as a factor that could increase the risk of CVD. Studies showed that a family history is associated with an increase in CVD mortality across long-term follow-up [33].

Secondly, the evaluation of dietary habits using the adherence score to the Saudi dietary guidelines revealed that fruit, olive oil, and non-alcoholic beer were more highly consumed in the non-CVD patients than in the CVD patients. These food items are rich in polyphenols and dietary fiber, nutrients for which high levels of intake were previously associated with a decreased risk of developing CVD [34]. Moreover, we found that the consumption of non-refined cereals and breads was particularly low relative to recommendation. The high intake of refined carbohydrate is reported to increase the risk of type 2 diabetes and CVD [35]. The Saudi dietary guidelines are well publicized; however, more effort may be needed on education and promotion of the guidelines to reduce the risk of diseases including CVD. 

Olive oil is the main source of fat in the Mediterranean diet and is linked with a lower mortality for CVD [18]. An olive-oil-rich diet is associated with enhanced lipoprotein metabolism and a reduction in oxidative damage, inflammation, blood pressure, endothelial dysfunction, and thrombosis [36]. A study by Guasch-Ferre et al, demonstrated that olive-oil intake, in particular the extra-virgin variety, decreased the risk of mortality and cardiovascular disease for individuals from Spain who were at high CVD risk [37]. Furthermore, a study by Carnevale et al. reported that olive oil advanced the post-prandial glucose and lipid profile in patients with impaired fasting glucose [38].

A study carried out by Woodside et al. found a strong correlation between the intake of fruits and vegetables and a reduction in the risk of developing coronary heart disease (CHD) [22]. Vegetables and fruits are a good source of nutrients, including vitamins, minerals, dietary fiber, and other biologically active compounds. These compounds have important mechanisms of action, including enhancing the immune system, reducing platelet aggregation, modulating cholesterol synthesis, reduction of blood pressure, and antioxidant, antibacterial, and antiviral effects [39]. 

The higher non-alcoholic beer consumption among the non-CVD patients may indicate that non-alcoholic beer could have a positive effect on heart health. It was reported that non-alcoholic beer can inhibit blood coagulation and platelet activation, which benefits the cardiovascular system without the negative effects of alcohol [32]. Despite the differences between non-CVD and CVD patients in their dietary habits, the lipid profile biomarkers, including total cholesterol, triglycerides, and LDL, were not significantly different. This is in contrast with the reported study by Rossouw, who showed that cholesterol levels are correlated with the risk of CHD, even at “normal” levels of cholesterol, in both men and women of all ages [40]. In addition, unpredictably, the levels of HDL were significantly higher in the CVD patients. The reason underlying this association is unknown, but one plausible explanation could be due to medications that were prescribed to CVD patients to control blood pressure, hyperlipidemia, and cardiac disease. These types of medications, such as niacin and atorvastatin, are known to enhance the lipid profile, and they were reported to increase HDL [41]. The current study also did not detect any significant differences between the non-CVD and CVD patients in the anthropometric measurements of weight, waist circumference, and BMI. This is in contrast with Alissa et al., who reported a strong significant relationship between BMI and the CVD risk in Saudi participants [29]. The inconsistency between these results could be due to differences in the sample size, which was smaller in this preliminary study. This study is the first to assess the adherence to the Saudi dietary guidelines among CVD male patients in Saudi Arabia. Currently, there is no validated food frequency questionnaire available specifically for the Saudi population; therefore, in this study, efforts were made to include food items that are more representative of the typical components of the Saudi diet. Furthermore, the analysis of adherence to the Saudi dietary guidelines was performed in parallel with the collection of blood biomarkers. As such, the data generated by this pilot study offer a unique insight into Saudi CVD populations, which may help in the planning and design of future studies to validate these findings. Further studies are now recommended to assess the association between the adherence to the Saudi dietary guidelines and the risk of CVD on a larger population sample. Moreover, as not all biomarker data of all for our patients were found in the patients’ electronic system, the sample size calculation for future studies needs to consider the missing biomarker data of patients in the hospital electronic records when determining the power of their studies to allow for examining a more complete dataset of biomarkers.

## 5. Conclusions

The data from this preliminary study report a number of significant differences between non-CVD and CVD patients in terms of intake of particular food groups and CVD family history. These factors could be important contributors to the CVD risk in the Saudi population. Further research is now needed, using a larger sample size, in order to validate these findings and increase insight into the risk factors of the Saudi lifestyle that are associated with CVD. 

## Figures and Tables

**Table 1:** The Saudi dietary guideline score.

No.	Food Groups	Frequency of Consumption (Servings/Month)					
		Never	1–4	5–8	9–12	13–18	>18
1	Non-refined cereals and bread ^a^	0	1	2	3	4	5
2	Fruit ^b^	0	1	2	3	4	5
3	Vegetable	0	1	2	3	4	5
4	Legumes	0	1	2	3	4	5
5	Fish	0	1	2	3	4	5
6	Olive oil	0	1	2	3	4	5
7	Non-alcoholic beer	0	1	2	3	4	5
8	Meat and meat products	5	4	3	2	1	0
9	Poultry	5	4	3	2	1	0
10	Full-fat dairy products	5	4	3	2	1	0
11	Sweets	5	4	3	2	1	0
12	Oils	5	4	3	2	1	0

^a^ Whole-grain bread, rice, pasta etc. ^b^ Fresh (e.g., apple, oranges, banana, grapes, etc.) and dried fruit, including dates.

**Table 2:** Demographic data of the study participants.

Demographic Variable		All Patients (*n* = 80)		Non-CVD Patients (*n* = 40)		CVD Patients (*n* = 40)		*p*-Value *
		*n*	%	*n*	%	*n*	%	
Age (years)	30–55	36.0	45.0	21.0	52.5	15.0	37.5	0.09
	56–80	44.0	55.0	19.0	47.5	25.0	62.5	
Marital Status	Married	73.0	91.3	34.0	85.0	39.0	97.5	0.05
	Single	7.0	8.8	6.0	15.0	1.0	2.5	
Education	None	6.0	7.5	1.0	2.5	5.0	12.5	0.13
	Elementary	12.0	15.0	7.0	17.5	5.0	12.5	
	Intermediate	8.0	10.0	2.0	5.0	6.0	15.0	
	High School	32.0	40.0	20.0	50.0	12.0	30.0	
	University	22.0	27.5	10.0	25.0	12.0	30.0	
Employment	Employed	34.0	42.5	13.0	32.5	21.0	52.5	0.07
	Non-Employed	46.0	57.5	27.0	67.5	19.0	47.5	

*n*: number of patients; CVD: cardiovascular disease. * *p*-value between non-CVD and CVD patients.

**Table 3:** Family and smoking history of the study participants.

Variables		All Patients (*n* = 80)		Non-CVD Patients (*n* = 40)		CVD Patients (*n* = 40)		*p*-Value *
		*n*	%	*n*	%	*n*	%	
Family History	Negative	54.0	67.5	32.0	80.0	22.0	55.0	0.02
	Positive	26.0	32.5	8.0	20.0	18.0	45.0	
Diabetic	Yes	36.0	45.0	9	22.5	27	67.5	0.06
	No	44.0	55.0	31	77.5	13	32.5	
Smoking	Never	29.0	36.3	18.0	45.0	11.0	27.5	0.2
	Former >3 years	28.0	35.0	11.0	27.5	17.0	42.5	
	Former <3 years	7.0	8.8	2.0	5.0	5.0	12.5	
	Current	16.0	20.0	9.0	22.5	7.0	17.5	

*n*: number of patients; CVD: cardiovascular disease. * *p*-value between non-CVD and CVD patients.

**Table 4:** Anthropometric measurements of the study participants.

Variables	All Patients		Non-CVD Patients		CVD Patients		*p*-Value *
	Median	IQR	Median	IQR	Median	IQR	
Height (cm)	168.0 (*n* = 80)	160.0–172.2	165.0 (*n* = 40)	160.0–170.8	170.0 (*n* = 40)	160.0–170.8	0.6
Weight (kg)	70.0 (*n* = 80)	68.0–84.0	75.0 (*n* = 40)	67.5–86.0	70.0 (*n* = 40)	68.8–80.0	0.4
BMI (kg/m^2^)	26.6 (*n* = 80)	23.4–29.4	27.2 (*n* = 40)	23.4–31.1	25.7 (*n* = 40)	23.4–27.7	0.3
WC (cm)	94.5 (*n* = 80)	88.2–99.7	95.0 (*n* = 40)	85.8–100.0	94.0 (*n* = 40)	90.0–98.3	0.5
Systolic BP	128.0 (*n* = 45)	117.5–140.0	128.0 (*n* = 26)	120.0–139.3	131.0 (*n* = 19)	119.5–142.0	0.1
Diastolic BP	76.0 (*n* = 45)	70.0–85.5	81.7 (*n* = 26)	75.0–87.8	70.0 (*n* = 19)	64.0–78.5	0.03
	**n**	**%**	**n**	**%**	**n**	**%**	
Underweight	7.0	8.8	2.0	5.0	5.0	12.5	
Normal weight	36.0	45.0	17.0	42.5	19.0	47.5	
Overweight	26.0	32.5	13.0	32.5	13.0	32.5	
Obese	11.0	13.8	8.0	20.0	3.0	7.5	

*n*: number of patients; CVD: cardiovascular disease; BMI: body mass index; BP: blood pressure; WC: waist circumference. * *p*-value between non-CVD and CVD patients.

**Table 5:** The score of the adherence to the Saudi dietary guidelines.

	All Patients (*n* = 80)		Non-CVD Patients (*n* = 40)		CVD Patients (*n* = 40)		*p*-Value *
	Mean	SD	Mean	SD	Mean	SD	
Non-refined cereals and bread	1.2	2.1	1.5	2.3	1.0	1.9	0.29
Fruit	4.9	0.4	5.0	0.0	4.8	0.5	0.02
Vegetable	4.9	0.5	5.0	0.0	4.9	0.7	0.15
Legumes	3.6	1.6	3.6	1.6	3.6	1.7	0.98
Fish	2.8	1.6	2.7	1.6	3.0	1.5	0.37
Olive oil	2.7	2.1	3.2	2.1	2.1	2.0	0.01
Non-alcoholic beer	0.8	1.5	1.3	1.8	0.4	0.9	0.01
Meat and meat products	1.5	1.4	1.5	1.4	1.5	1.5	0.93
Poultry	1.5	1.6	1.2	1.5	1.8	1.6	0.06
Full-fat dairy products	0.1	0.6	0.1	0.3	0.2	0.7	0.17
Sweets	2.5	2.1	2.4	2.2	2.7	2.1	0.76
Oils	0.7	1.7	0.7	1.8	0.7	1.7	0.80
Total adherence to Saudi dietary guidelines	27.3	6.0	28.1	6.6	26.5	5.4	0.23

CVD: cardiovascular disease. * *p*-value between non-CVD and CVD patients.

**Table 6:** The association between different clinical and anthropometric factors (dependent) and total adherence to Saudi dietary guideline score (independent). Results of multiple linear regression analysis.

	*β*-Coefficient ± SE	*p*-Value
Model 1: Systolic blood pressure	−0.284 ± 0.508	0.579
Model 2: Serum cholesterol	−0.071 ± 0.023	0.004
Model 3: Low-density lipoprotein	−0.072 ± 0.028	0.012
Model 4: High-density lipoprotein	−0.021 ± 0.011	0.074
Model 5: Triglycerides	−0.032 ± 0.018	0.077
Model 6: Waist circumference	−0.308 ± 0.229	0.183
Model 7: Body mass index	−0.087 ± 0.102	0.399

All models were adjusted for age, smoking history, educational level, diabetic history, and family history of CVD. SE: Standard error.

**Table 7:** Cardiovascular-related biomarkers.

Biomarkers	All patients		Non-CVD Patients		CVD Patients		*p*-Value *
	Median	IQR	Median	IQR	Median	IQR	
**Lipids**							
TC (mmol/L)	4.0 (*n* = 80)	3.0–4.6	4.0 (*n* = 40)	3.4–4.6	3.7 (*n* = 40)	3.0–4.6	0.7
TG (mmol/L)	1.3 (*n* = 80)	0.8–1.7	1.3 (*n* = 40)	0.8–1.9	1.2 (*n* = 40)	0.9–1.6	0.5
LDL (mmol/L)	2.7 (*n* = 46)	1.9–3.4	3.0 (*n* = 18)	2.3-3.6	2.6 (*n* = 28)	1.9-3.2	0.2
HDL (mmol/L)	1.1 (*n* = 42)	0.8–1.3	1.0 (*n* = 17)	0.6–1.1	1.1 (*n* = 25)	1.0–1.5	0.03
**Hematological**							
FBG (mmol/L)	6.5 (*n* = 80)	5.5–9.6	6.1 (*n* = 40)	5.1–7.2	7.5 (*n* = 40)	6.2–11.7	0.006
HbA1c (mmol/L)	7.4 (*n* = 80)	5.8–8.9	6.3 (*n* = 40)	5.4–7.4	8.2 (*n* = 40)	6.1–9.0	0.1
HGB (g/dl)	12.0 (*n* = 80)	10.0–13.6	12.3 (*n* = 40)	9.0–14.0	12.0 (*n* = 40)	10.3–13.1	0.7
HCT (%)	35.4 (*n* = 80)	30.1–40.5	36.5 (*n* = 40)	27.5–41.0	35.2 (*n* = 40)	31.8–39.3	0.8
**Electrolytes**							
Na (mmol/L)	137.0 (*n* = 80)	134.0–139.0	138.0 (*n* = 40)	135.0–140.0	137.0 (*n* = 40)	131.0–139.0	0.9
K (mmol/L)	3.9 (*n* = 80)	3.5–4.2	3.7 (*n* = 40)	3.5–4.0	4.1 (*n* = 40)	3.5–4.4	0.03
Cl (mmol/L)	99.5 (*n* = 80)	97.0–103.0	101.0 (*n* = 40)	98.0–104.0	99.0 (*n* = 40)	94.8–102.0	0.05
**Cardiac Enzymes**							
AST (U/L)	27.0 (*n* = 69)	19.0–41.3	26.0 (*n* = 29)	19.0–45.0	28.5 (*n* = 40)	19.0–40.5	0.12
CK (IU/L)	109.0 (*n* = 52)	52.0–214.0	95.5 (*n* = 12)	49.5–171.8	116.0 (*n* = 40)	63.0–249.0	0.4
LDH (U/L)	244.5 (*n* = 51)	191.8–304.0	215.0 (*n* = 11)	176.5–250.0	255.0 (*n* = 40)	212.0–310.0	0.1
CTN–I (ug/L)	0.1 (*n* = 49)	0.0–0.6	0.1 (*n* = 09)	0.0–0.3	0.1 (*n* = 40)	0.0–0.6	0.4

TC = total cholesterol; TG = triglycerides; LDL = low-density lipoprotein; HDL = high-density lipoprotein; FBG = fasting blood glucose; HbA1 = glycated hemoglobin; HGB = hemoglobin; HCT = hematocrit; Na = sodium; K = potassium; Cl = chloride; AST = aspartate aminotransferase; CK = creatine kinase; LDH = lactate dehydrogenase; CTN-I = cardiac troponin I. * *p*-value between non-CVD and CVD patients.
